# The Presence of Norovirus and Adenovirus on Environmental Surfaces in Relation to the Hygienic Level in Food Service Operations Associated with a Suspected Gastroenteritis Outbreak

**DOI:** 10.1007/s12560-017-9291-7

**Published:** 2017-03-15

**Authors:** Leena Maunula, M. Rönnqvist, R. Åberg, J. Lunden, M. Nevas

**Affiliations:** 1 The Department of Food Hygiene and Environmental Health, Faculty of Veterinary Medicine, University of Helsinki, P.O. Box 66, Agnes Sjöberginkatu 2, 00014 Helsinki, Finland; 2 Risk Assessment Research Unit, Finnish Food Safety Authority Evira, Mustialankatu 3, 00790 Helsinki, Finland; 3 Food Safety Department, Environment Centre, Helsinki, Finland

**Keywords:** NoV, AdV, Food service operation, Foodborne outbreak, Hygiene inspection, Environmental sampling

## Abstract

Norovirus (NoV) gastroenteritis outbreaks appear frequently in food service operations (FSOs), such as in restaurants and canteens. In this study the presence of NoV and adenovirus (AdV) genomes was investigated on the surfaces of premises, especially in kitchens, of 30 FSOs where foodborne gastroenteritis outbreaks were suspected. The objective was to establish a possible association between the presence of virus genomes on surfaces and a visual hygienic status of the FSOs. NoV genome was found in 11 and AdV genome in 8 out of 30 FSOs. In total, 291 swabs were taken, of which 8.9% contained NoV and 5.8% AdV genome. The presence of NoV genomes on the surfaces was not found to associate with lower hygiene level of the premises when based on visual inspection; most (7/9) of the FSOs with NoV contamination on surfaces and a completed evaluation form had a good hygiene level (the best category). Restaurants had a significantly lower proportion of NoV-positive swabs compared to other FSOs (canteens, cafeteria, schools etc.) taken together (*p* = 0.00014). The presence of a designated break room for the workers was found to be significantly more common in AdV-negative kitchens (*p* = 0.046). Our findings suggest that swabbing is necessary for revealing viral contamination of surfaces and emphasis of hygiene inspections should be on the food handling procedures, and the education of food workers on virus transmission.

**Electronic supplementary material:**

The online version of this article (doi:10.1007/s12560-017-9291-7) contains supplementary material, which is available to authorized users.

## Introduction

Human noroviruses (NoVs) are the most common causative agents for viral gastroenteritis in developed countries (Tam et al. 2012; Robilotti et al. 2015). Their main transmission route is from person to person, especially during the annual epidemiological peak season. Recently, it was estimated that globally about 14% of NoV infections are linked to food, based on data over 10 years (Verhoef et al. 2015). About 59% of all foodborne illnesses are caused by NoV (Scallan et al. 2011). Food can be contaminated through water-containing human faecal material or sewage, but very commonly foodborne outbreaks are transmitted by infected food handlers during food handling directly or through contaminated fomites (Todd et al. 2008; Rönnqvist et al. 2014; Grove et al. 2015).

Foodborne outbreaks often occur in food service operations (FSOs), such as in restaurants. In the U.S. 66% of gastroenteritis outbreaks occurred in restaurants in 2006–2007 (Gould et al. 2013). According to a survey by Carpenter et al. (2013), nearly 60% of food workers from 391 restaurants claimed that during the previous year they had worked while they were ill and 20% reported that they had had gastroenteritis or they had vomited during work shifts. The personnel infected with NoV may have been working while ill due to rapid onset of the disease, or as asymptomatic carriers, because excretion of NoV continues after disappearance of the symptoms (Atmar et al. 2008). In a Danish study, food handlers were asymptomatic during food handling in the majority of the outbreaks involving food handlers (Franck et al. 2015).

NoV, non-enveloped ssRNA viruses that belong to the *Caliciviridae* family express vast genetic variation (Robilotti et al. 2015). Human NoV infections are mainly caused by genogroup I and II viruses, and genotype GII.4 with its evolving variants are especially efficient in transmitting itself via food handlers. NoV transmits efficiently, since low virus doses are enough to cause infection and since persons of all ages can be susceptible. NoV are persistent, as exemplified by the cultivable NoV surrogates, feline caliciviruses, and murine NoVs, that survive on environmental surfaces for 7 days (D’Souza et al. 2006; Mormann et al. 2015). In recent studies the virus genome has been successfully demonstrated in swabs taken from inanimate surfaces of premises handling food (Boxman et al. 2011; Rönnqvist et al. 2013).

Adenoviruses (AdV), a manifold group of viruses, are common causative agents of enteric infections (Rodriguez-Lazaro et al. 2012), often giving symptoms in children, but being asymptomatic in adults. While many AdV types attack the upper respiratory tract or eyes, especially types 40 and 41 cause gastroenteritis. High AdV titres are constantly present in sewage due to their high prevalence in populations and thus have been proposed as a marker for faecal contamination in water (Wyn-Jones et al. 2011; Bofill-Mas et al. 2006).

Based on the EC Regulation 852/2004, the food business operators are responsible for the safety of the food produced, but the compliance of their actions with the regulations is controlled by food safety authorities (Anon 2004). Inspections of food premises may be considered the main tool for official food controllers to ensure that the actions of food business operators are consistent with the regulations.

Currently there have only been a few published studies (Boxman et al. 2011; Verhoef et al. 2013; Boxman et al. 2015), which describe the relationship between the presence of viral foodborne pathogens and the hygiene level of FSOs. The objective of our study was to survey the presence of NoV and AdV genomes on the surfaces of food providing premises linked to suspected foodborne gastroenteritis outbreaks. AdV analysis was included in order to further assess, how successful was sampling and transportation. In addition, we evaluated whether the hygiene level of the premises would be associated with virus contamination and/or reveal any contributing factors to the presence of NoV or AdV contamination in the FSOs.

## Materials and Methods

### Description of Settings and Sampling

In total, 30 FSOs in which a suspicion of foodborne gastroenteritis outbreak was reported (an obligatory reporting system in Finland) were included in the study. There were 8, 15 and 7 FSOs, respectively, during 2012–2014. All but two located in the metropolitan area of Helsinki (all located in southern Finland). Local food inspectors visited the FSOs immediately after the suspected foodborne gastroenteritis outbreak had been reported as part of their routine inspection. For this study, they took 4–15 (median 10) swabs from the surfaces of the premises for viral genome detection tests and completed an evaluation questionnaire. The information about what pathogen eventually caused the suspected outbreaks and whether the outbreaks were foodborne or not, is out of scope of the present study.

In total, 291 swabs were taken from the surfaces of kitchens, break rooms and staff toilet facilities according to the sampling scheme. Swabs were taken from six sites (cutting board, table surface, knife/ladle, door handle of refrigerator, cold drawers and one optional surface) in the kitchen and four sites among six (microwave oven handle, handle of refrigerator, toilet door handles, toilet tap handle, toilet light switcher, and one optional surface) in the break room-toilet areas according to what was available to be sampled on the sites. Some modifications to the scheme were allowed depending on the setting. In addition, 25 completed evaluation questionnaires were received (see hygiene-level evaluation). The personnel consisted of <10 persons in 15 FSOs, ≥10 persons in 3 FSOs, and personnel size was unknown in 7 FSOs. Four FSOs reported having new work force and 7 temporary work force.

### Viral Nucleic Acid Detection and Genotyping

NoV and AdV detection and genotyping was performed according to a detailed description of Oristo et al. (2016) and Rönnqvist et al. (2013). In brief, swabbing was performed with polyester swabs (175KS01, Mekalasi Oy, Helsinki, Finland) moistened with phosphate buffered saline (PBS, pH 7). The recommended swabbing area was 5 cm × 5 cm, except on those occasions where it was not technically possible, such as a door handle. After swabbing, the swabs were transported to the virus laboratory at the University of Helsinki in 2 ml of PBS within 24 h. After semi-direct lysis of viral material (lysis is performed in the presence of swab still in the tube; Rönnqvist et al. 2014) from swabs nucleic acid was extracted with the Nuclisens kit (Biomerieux). QuantiTect probe RT-PCR and QuantiTect probe PCR kits were employed for gene amplification using degenerate primers and a labelled Taqman probe for genogroup I and II separately targeted for the NoV polymerase-capsid gene junction (Loisy et al. 2005; Rönnqvist et al. 2013) and hexon gene of AdV (Jothikumar et al. 2005) in a qualitative manner, respectively.

AdV DNA containing samples were further tested with qPCR with 40/41 specific primers and a Taqman probe (van Maarseveen et al. 2010). Primers described by Vinje et al. (2004) were used for genotyping of NoV-positive findings for the polymerase gene region using a one-step RT-PCR kit (Qiagen). For capsid gene region, specific primer pair for GII NoV was used for double PCR (Schultz et al. 2011). For details of AdV and NoV genotyping, see Oristo et al. (2016). The amplicons were analysed by a nucleic acid sequencing service. If other genotyping methods failed, specific NoV GII.4 primers were applied with SYBR green chemistry (Maunula et al. 2009).

### Hygiene-Level Evaluation

In total, 34 items asked in the questionnaires (Table S1) were evaluated by the inspectors during visual inspection. The hygiene level at the FSOs was evaluated by the professional food inspectors, using a form with four-point evaluation scale (1 = Good, 2 = Satisfactory, 3 = Passable, 4 = Poor) for 21 items and “yes/no” answers for 12 items. In addition, the size of the staff was asked for. The sites inspected included the kitchens, the customer areas, and the storage rooms for cleaning equipment. Checks were also made to determine whether a separate social room and/or separate toilet was available for the personnel, and whether the existing toilets were equipped with automatic faucets. We established whether there were any new or leased personnel at the settings, or if the personnel or their family members had been ill recently. Concerning the handling of the foods, the appropriateness of possible chilling and re-heating the foods was evaluated, as well as the possible use of imported frozen berries. The traceability of the foods was also assessed.

Of the 34 questions, 22 concerning hygiene were selected and they were included in the scoring to determine the hygiene level or category. The values obtained from 22 main specific items, 18 rated with 1–4 (see above), and the 4 dichotomous items received 1 or 2 (yes = 1, no = 2). The hygiene score was the sum of the values. If an FSO had received all the best values (value 1), its score was 18 plus 4, summing up to 22. Each category was determined to have a range of 10 points starting from the higher hygiene level (scores in the best category: 22–31 points, the second category: 32–41, the third category: 42–51) to the lower hygienic level (as an exception, the scores 52–80 belonged to the fourth category). The four categories were “Good”, “Satisfactory”, “Passable” and “Poor” hygiene level.

### Statistical Analyses

Differences in the hygiene evaluation results measured on a four-grade scale between the NoV- or AdV-positive and NoV- or AdV-negative kitchens were analysed with the Mann–Whitney test. The relationship between factors that were measured on a dichotomous scale (yes/no) with presence of NoV or AdV was analysed with Fisher’s exact test. The statistical degree of confidence was set at a level of 95% (*p* < 0.05).

## Results

### Noro- and Adenovirus Findings on Environmental Surfaces

The 30 FSOs associated with suspected foodborne gastroenteritis outbreaks were sampled for the presence of NoV and AdV genome. More visits were made to FSOs between January and June than between July and December (21 vs 9 visits). In all, virus genome was present on environmental surfaces in 18 (60.0%) out of 30 FSOs. NoV genome was detected on surfaces in 11 (36.7%) and AdV genome in 8 (26.7%) of the FSOs (Table 1); 6 and 5 kitchens were contaminated by NoV and AdV, respectively. Both of the viruses were found once in the same FSO. About equal proportion of FSOs had NoV contamination between January and June than July and December (8/21 vs 3/9).

**Table 1:** Distribution of NoV-positive and AdV-positive environmental surface samples over different types of food service operations (FSOs)

Type of food premise^a^	FSOs no. NoV-positive/total (%)	Samples no. NoV-positive/total (%)	FSOs no. AdV-positive/total (%)	Samples no. AdV-positive/total (%)
Restaurant	2/14 (14.3)	3/134 (2.2)	3/14 (21.4)	11/134 (8.2)
Canteen	3/5	6/46 (13.0)	1/5	1/46 (2.2)
Cafeteria	2/3	10/26 (38.5)	0/3	0/26 (0)
School	2/3	4/38 (10.5)	1/3	1/38 (2.6)
Day care	1/2	1/15 (6.7)	1/2	1/15 (6.7)
Course centre, spa	1/3	2/32 (6.3)	2/3	3/32 (9.4)
Total	11/30 (36.7)	26/291 (8.9)	8/30 (26.7)	17/291 (5.8)

^a^Swab samples taken mainly from kitchens, break rooms and sanitary areas

NoV genome was detected in 26/291 (8.9%) and AdV genome in 17/291 (5.8%) of swabs, almost exclusively in separate samples. NoV genome was more often detected in swabs taken from canteens, cafeteria or schools (10.5–38.5%) than in swabs from restaurants (2.2%). Restaurants that comprised 46.7% of all the settings in this study had a significantly lower proportion of NoV-positive swabs compared to other settings taken together (*p* = 0.00014) (Table 1). In contrast, AdV genome was commonly found in swabs taken from restaurants (8.2%), with no significant difference in proportion of AdV-positive swabs between restaurants and other settings.

The occurrence of NoV contamination was evenly distributed in interiors of FSOs, in 6 out of 30 kitchens, in 2 out of 9 break rooms and in 5 out of 25 staff toilets, ranging between 20 and 22% (data not shown). However, the frequency of NoV-positive swabs taken from different premises ranged between 6.0 and 16.7%, with fewer NoV-positive swabs in kitchens than in break rooms and toilet facilities (Table 2). The number of NoV-positive swabs taken from kitchens was significantly lower than their number taken from sanitary facilities (*p* = 0.025). AdV contamination showed comparable distribution (kitchen 5/30, break room 1/9 and toilets 3/25) to NoV, but somewhat less frequently (11–17%).

**Table 2:** Distribution of NoV and AdV findings over targeted surfaces

	NoV	%	AdV	%
Kitchen				
Cutting board	0/23	0.0	1/23	4.3
Table surface	2/32	6.3	3/32	6.3
Knife/ladle	1/22	4.5	2/22	9.4
Door handle refrigerator or freezer	3/30	10.0	0/30	0
Cold drawer	0/12	0.0	2/12	16.7
Handwashing facilities	3/25	12.0	2/25	8.0
Other surface	1/22	4.5	0/22	0
Total	10/166	6.0	10/166	6.0
Break room				
Microwave oven	1/3	33.3	0/3	0
Door handle refrigerator or freezer	1/3	33.3	1/3	33.3
Other surface	0/6	0.0	0/6	0.0
Total	2/12	16.7	1/12	8.3
Staff toilet				
WC door handle	3/24	12.5	3/24	12.5
WC tap	2/25	8.0	1/25	4.0
WC light switches	3/17	17.6	0/17	0
Other surface	3/5	60.0	1/5	20.0
Total	11/71	15.5	5/71	7.0
Public (customer) area or toilet	3/42	7.1	1/42	2.4
Total	26/291	8.9	17/291	5.8

One of the two viruses was found at least once on each targeted swabbing surfaces, when all FSOs were taken into account (Table 2), NoV contamination on 12 and AdV on 10 out of 15 targeted surfaces. NoV genome was detected often on light switches or on door handles of toilets. Furthermore, handwashing facilities or door handles of refrigerators/freezers, but not cutting boards, were contaminated in kitchens. Virus genome was also found on microwave ovens and door handles of break rooms.

All NoV findings were of genogroup GII. Eleven viruses were typed: Five were GII.4 and one GII.1 (a day care centre), typing wasn’t successful in 5 samples due to low virus genome content in the swab. Enteric AdV genome type 40/41 was found in three AdV containing samples, all taken from one restaurant, the rest AdV-positive samples were not of type 40/41. No attempts were made to determine other AdV genotypes.

### Hygienic Inspection: Hygienic Levels on the Premises

None of the specific factors scored at the hygiene evaluations associated with FSOs with NoV contamination on surfaces based on the 25 questionnaires completed during visual hygienic inspection (Table 3). The presence of a designated break room for the workers, however, was significantly more common in FSOs without AdV contamination on surfaces (*p* = 0.046). Also a separate toilet for the kitchen workers and the customers was more common in those FSOs, although the difference was not significant (*p* = 0.073). Automatic handwashing taps in the kitchen or the toilets, the use of frozen berries of foreign origin or heating/not heating the berries were not observed to influence the likelihood of the presence of NoV or AdV genome on surfaces. NoV contaminated FSOs had about two times more workers than non-NoV-contaminated FSOs (7.14 vs 3.63; *p* > 0.05), whereas AdV contaminated FSOs had less workers (2.60 vs 5.88; *p* > 0.05), but differences were not significant. Temporality of workers or presence of new workers did not influence the likelihood of viral contamination on surfaces. Unexpectedly, even knowledge about gastroenteritis among any workers prior to suspicion of an outbreak in these FSOs didn’t predict the presence of NoV or AdV on surfaces.

**Table 3:** Hygienic evaluation of FSOs with or without NoV or AdV contamination on surfaces (*n* = 25)

Evaluated factor	Hygiene evaluation mean^a^					
	Norovirus			Adenovirus		
	Positive	Negative	*p* value^b^	Positive	Negative	*p* value^b^
Condition and cleanliness of kitchen	1.3	1.6	<0.05	1.4	1.6	<0.05
Suitability of kitchen for the activities	1.2	1.3	<0.05	1.4	1.2	<0.05
Adequacy of handwashing sites	1.1	1.3	<0.05	1.2	1.3	<0.05
Food serving conditions	1.2	1.2	<0.05	1.3	1.1	<0.05
Adequacy of cleaning equipment	1.7	1.6	<0.05	1.4	1.7	<0.05
Cleanliness of employee break room	1.4	1.7	<0.05	1.0	1.7	<0.05
Adequacy of work clothing	1.4	1.5	<0.05	1.5	1.4	<0.05
Total	1.3	1.5		1.3	1.4	

^a^The evaluation was done using the following grading_ 1 = Good, 2 = Satisfactory, 3 = Passable, 4 = Poor

^b^Mann–Whitney test

The hygienic scores for 25 evaluated FSOs ranged between 23 and 50, where 22 was the best and 80 the worst possible score based on 22 specific items (Table 4). More than half (14/25) of the FSOs had good hygiene levels, and most (7/9) of the FSOs with NoV contamination on surfaces belonged to this category. Of the four settings that had only passable hygiene scores, one cafe had NoV contamination on its premises (score 45). Most FSOs with AdV contamination on surfaces were of good hygiene (4/6). The above-mentioned restaurant with enteric AdV 40/41 genome on its premise’s surfaces scored 50 (passable hygiene level) that was the worst value received in this study.

**Table 4:** The presence of NoV and AdV contamination on environmental surfaces in FSOs (*n* = 25) scored by their hygiene levels

Hygiene category	NoV+ on surfaces	AdV+ on surfaces
Good hygiene (score^a^ 22–31)	7/14 (50.0%)	4/14 (28.6%)
Satisfactory hygiene (32–41)	1/8 (12.5%)	1/8 (12.5%)
Passable hygiene (42–51)	1/3 (33.3%)	1/3 (33.3%)
Poor hygiene (52–80)^b^	0/0	0/0
Total	9/25	6/25

^a^Range of scores 22–80, based on 22 criteria

^b^All lower scores are combined, since there are no FSOs in these categories

## Discussion

We investigated the presence of viruses on the surfaces of premises and concomitantly evaluated hygiene level in FSOs where foodborne gastroenteritis outbreaks had been suspected. In this study especially NoV, but also AdV genomes were frequently detected on environmental surfaces, also on the kitchen premises. NoV contamination often occurred also in FSOs that were evaluated as having the best hygiene level. In this study the low number of FSOs hampered statistical analyses. The study showed, however, that viral swab analyses could give information that was not obvious by visual hygiene inspection.

In the comprehensive study of Boxman et al. (2011), swabbing the surfaces on the premises of catering companies revealed NoV genome in 4.2% of the companies in general and in 61.1% of the companies associated with gastroenteritis outbreaks. The results are in good agreement with our value of 36.7% in FSOs with suspected foodborne outbreaks. Factors affecting the prevalence are, among others, features of the swabbing method used, number of swabs taken and the inclusion of high-risk institutions, such as retirement homes. In the study of Boxman et al. (2011) in addition to retirement homes, also canteens were shown to be at risk for NoV gastroenteritis outbreaks. Although the number of canteens in our study was limited, NoV contamination was observed in several of them as well as in other non-restaurant FSOs. It seems that restaurants cope better with virus risk than other FSOs do. The vulnerability of canteens, cafes and school canteens for NoV contamination might be connected to periods of over-crowdedness at the premises. People visit those FSOs on every working/school day, while restaurants have a different visiting pattern. In the restaurants of our study, protection of served food was found to be a more common habit than in other FSOs (data not shown). Employing buffet service typical for canteens rather than table service may also affect the likelihood of virus transmissions.

The study demonstrated the presence of NoV genome on surfaces that are commonly touched and not often cleaned, such as door handles, whereas NoV was not found for example on regularly washed cutting boards. NoV by handwashing facilities in the kitchen may indicate that viruses have been removed from the hands by handwashing, but virus-contaminated door handles of fridges and freezers clearly reveal inadequate hygienic practices among kitchen staff. In general, the results are in line with those reported by Rönnqvist et al. (2013) that demonstrated the presence of NoV on surfaces, such as on coffee machines, in break rooms. The significantly lower prevalence in kitchens than in sanitary facilities that we observed in our study was also found by Boxman et al. (2015) in hospital central kitchens and non-hospital health care settings, but not in catering companies.

Swab testing is suitable for revealing signs of contamination that NoV excreting persons have left on the surfaces of the premises, but it is less suitable for revealing NoV in food, such as leafy green, oysters or frozen berries, that arrived in a kitchen already contaminated. However, the role of food handlers in causing foodborne NoV outbreaks is indeed prominent, as exemplified in a study of Hedberg et al. (2006), in which the handling of food by an infected person or carriers was identified as a most common (65%) contributing factor in foodborne outbreaks. In our study, a conclusion can be drawn based on the viral contamination of surfaces that person(s) having NoV infection had likely visited the premises or was/were working in them in about one third of the FSOs.

Although the relation between viral swab results and visual hygiene inspection has been rarely investigated, other microbial swabs have been taken during inspections. In a study by Kassa et al. (2001) the presence of enteric bacteria, but not of pathogenic bacteria, was reported on environmental surfaces of mainly low-category FSOs but no direct correlation existed between microbial swab findings and rating scores based on visual inspection. This lack of correlation agrees with our observations concerning NoV contamination.

Our study was focused on NoV, but AdVs were also included to demonstrate the performance of the swabbing method, as it was expected to be more common than NoV (Maunula et al. 2013). AdV on surfaces may also originate from the respiratory track (Oristo et al. 2016), which may explain why AdV, although present frequently, was not detected in the same FSOs as NoV, which is usually of faecal origin. Another reason might be the fact that NoV, but not AdV, shows a clear seasonality. Interestingly, enteric AdV 40/41 type was found in one restaurant with low hygiene procedures, but more studies are needed to find out, whether enteric AdV could serve as a marker for faecal contamination.

In our study, we found that separate social rooms and toilets for staff were more common in AdV-negative than AdV-positive kitchens, whereas Boxman et al. (2011) found that NoV was detected more frequently in restaurants that had separate staff bathrooms. The possibility for the staff to have breaks and meals in break rooms instead of kitchens may be reflected in less frequent virus contamination of kitchen surfaces, which would explain our results. On the other hand, it is more likely to detect NoV on break room than on kitchen surfaces, since gloves are not used there, which may partly explain the results of Boxman et al. (2011).

It has been reported that it is especially challenging to use short duration visual inspection to monitor faults in staff personal hygiene (Leisner et al. 2014). Petran and colleagues (Petran et al. 2012) could find correlations between hygienic level and outbreaks in restaurants when outbreaks caused by NoV, Clostridium or Salmonella were investigated based on as many as 54 criteria. According to them, 50% of the relevant criteria were related to food handling, 37% to facilities and 15% to personal hygiene. Verhoef et al. (2013) found that knowledge of NoV was low especially in catering companies as compared to institutional settings when they observed gaps in education. The instructions concerning cleaning procedures for restaurants or the education of food handlers were not evaluated in our study. An open question remains, whether more detailed inspection than performed here, could have revealed food safety violations typically leading to NoV outbreaks. Thus, more extensive studies are needed in future.

In future, efforts should be made to encourage taking swabs if possible before cleaning in suspected outbreak situations to reveal surface contamination, as reviewed by Tebbutt (2007). More rapid swabbing tests are needed to provide time to take steps to prevent virus transmission via fomites. Strategies for how to influence personal hygiene practices among staff should be developed. Hygiene practices such as handwashing routines and the use of gloves should be evaluated during inspections. Education according to hygiene guidelines that take into account virus risks (Codex alimentarius, FAO/WHO, 2012) is recommended.

## Electronic supplementary material

Below is the link to the electronic supplementary material.

Supplementary material 1 (DOCX 113 kb)
