# Supplementary material for: The Presence of Norovirus and Adenovirus on Environmental Surfaces in Relation to the Hygienic Level in Food Service Operations Associated with a Suspected Gastroenteritis Outbreak
Source: Food Environ Virol. 2017 Mar 15;9(3):334–41. doi: 10.1007/s12560-017-9291-7 (PMC5548845; doi:10.1007/s12560-017-9291-7)
Supplement: Supplementary file 1 — Supplementary material 1 (DOCX 113 kb) [file 12560_2017_9291_MOESM1_ESM.docx]

**Table S1** Content of the hygiene evaluation questionnaire.

|  | Question | Evaluation |
| --- | --- | --- |
|  | **Cooking facilities** |  |
| 1 | General cleanliness and order of the whole operation of the premises | (1-4) |
| 2 | Size of facilities compared to scale of activity | (1-4) |
| 3 | Separation of treatment of mature and raw foods | (1-4) |
| 4 | Adequacy of hand-washing points with respect to the activity | (1-4) |
| 5 | Hand washing facility is equipped with detergent dispenser and hand towel paper | (1-4) |
| 6 | Hand washing facility has an automatic faucet | (yes, no) |
| 7 | Use of protective gloves | (1-4) |
| 8 | Condition and cleanability of working surfaces, equipment and working tools | (1-4) |
| 9 | Cleanliness of working surfaces, equipment and working tools | (1-4) |
| 10 | Condition and cleanability of ceiling, floor and wall surfaces | (1-4) |
| 11 | Cleanliness of ceiling, floor and wall surfaces | (1-4) |
|  | **Facilities for food service and customers** |  |
| 12 | General cleanliness and order | (1-4) |
| 13 | Appropriateness of catering equipment for warm products | (1-4) |
| 14 | Appropriateness of catering equipment for cold products | (1-4) |
| 15 | Protection of served foods | (1-4) |
|  | **Cleaning equipments** |  |
| 16 | General cleanliness and order | (1-4) |
| 17 | Condition and cleanliness of kitchen cleaning tools | (1-4) |
|  | **Staff facilities** |  |
| 18 | Staff facilities have a separate social space | (yes, no) |
| 19 | Personnel has a separate toilet | (yes, no) |
| 20 | Staff toilets have automatic faucets | (yes, no) |
| 21 | General cleanliness and order | (1-4) |
|  | **Staff** |  |
| 22 | The number of staff (in the kitchen) | (number) |
| 23 | Are there new employees? | (yes, no) |
| 24 | Is there temporary employment? | (yes, no) |
| 25 | Is any of the staff or the family members of the staff sick? | (yes, no) |
| 26 | Is the documentation concerning the state of health of the staff updated? | (yes, no) |
| 27 | Does the staff working in the kitchen wear special clothing, jewelery, watches and clocks | (1-4) |
|  | **Handling and traceability** |  |
| 28 | Food dishes are cooled in the premises | (yes, no) |
| 29 | Appropriateness of food chilling | (1-4) |
| 30 | Dishes are re-heated in the premises | (yes, no) |
| 31 | Appropriateness of food re-heating | (1-4) |
| 32 | Frozen berries are used in the food preparation in the premises | (yes, no) |
| 33 | Foreign frozen berries are heated before use | (yes, no) |
| 34 | Traceability of food products | (1-4) |

Scoring for hygiene level categories included questions 1-21 and 27.
